# A Framework of Combining Short-Term Spatial/Frequency Feature Extraction and Long-Term IndRNN for Activity Recognition †

**DOI:** 10.3390/s20236984

**Published:** 2020-12-07

**Authors:** Beidi Zhao, Shuai Li, Yanbo Gao, Chuankun Li, Wanqing Li

**Affiliations:** 1 Glasgow College, University of Electronic Science and Technology of China, Chengdu 611731, China; beidizhao@hotmail.com; 2 School of Control Science and Engineering, Shandong University, Jinan 250061, China; 3 School of Software, Shandong University, Jinan 250061, China; ybgao@sdu.edu.cn; 4 School of Information and Communication Engineering, North University of China, Taiyuan 030051, China; chuankun@nuc.edu.cn; 5 School of Computing and Information Technology, University of Wollongong, Wollongong 2522, NSW, Australia; wanqing@uow.edu.au

**Keywords:** IndRNN, activity recognition, SHL dataset, smartphone sensors

## Abstract

Smartphone-sensors-based human activity recognition is attracting increasing interest due to the popularization of smartphones. It is a difficult long-range temporal recognition problem, especially with large intraclass distances such as carrying smartphones at different locations and small interclass distances such as taking a train or subway. To address this problem, we propose a new framework of combining short-term spatial/frequency feature extraction and a long-term independently recurrent neural network (IndRNN) for activity recognition. Considering the periodic characteristics of the sensor data, short-term temporal features are first extracted in the spatial and frequency domains. Then, the IndRNN, which can capture long-term patterns, is used to further obtain the long-term features for classification. Given the large differences when the smartphone is carried at different locations, a group-based location recognition is first developed to pinpoint the location of the smartphone. The Sussex-Huawei Locomotion (SHL) dataset from the SHL Challenge is used for evaluation. An earlier version of the proposed method won the second place award in the SHL Challenge 2020 (first place if not considering the multiple models fusion approach). The proposed method is further improved in this paper and achieves 80.72% accuracy, better than the existing methods using a single model.

## 1. Introduction

Human activity recognition has been an active research area for decades and has many practical applications such as in video surveillance [1,2,3], human–computer interaction [4] and gaming [5]. With the ubiquity and portability of mobile phones and the development of mobile phone sensors, there has been a growing interest in smartphone-sensors-based human action recognition [5,6,7,8,9]. Applications of smartphone-sensors-based activity recognition for indoor localization [8], real-time smartphone activity classification [9] and transportation recognition [7] have been actively investigated.

Different from the conventional video-based human action recognition [10], the data captured from smartphone sensors show some specific characteristics. For example, due to the mechanism of smartphone sensors, it has been shown [11] that the data are of a periodic nature. Moreover, the sampling rate of smartphone sensors is usually high, resulting in a large amount of long-range data. Furthermore, different users have different living habits, and people usually place their mobile phones in different locations on their bodies, which causes large differences in the distribution of data. The locations of the sensors/smartphone change over time and with different actions, although they are fixed to certain locations on the body. For example, the smartphone is fixed on the hand, but it moves with the hand’s movement. In addition to the large variance of data, the activity categories used in the smartphone-sensors-based classification are also different from those of conventional human action recognition. Besides the locomotion of a person, the transportation mode is also considered an important classification task, including taking a car, bus, train and subway, which could be confusing.

To prompt the development of smartphone-sensors-based activity recognition, the Sussex-Huawei Locomotion (SHL) Challenge [12] has been organized for three years from 2018 to 2020. It is based on the large-scale SHL dataset recorded over seven months by three participants engaging in eight transportation activities in real-life settings, including Still, Walk, Run, Bike, Car, Bus, Train and Subway [13]. This year’s edition (2020) of the challenge [12] aims to realize the user independence and location independence.

In some works in the literature, authors proposed the use of methods such as conventional handcrafted-features-based methods and deep-learning-based methods for smartphone-sensors-based activity recognition. Especially with the rapid development of deep learning, many convolutional-neural-network (CNN)-based methods and recurrent-neural-network (RNN)-based methods have been developed in the last few years. For the CNN-based methods, EmbraceNet [14] and DenseNet [15] have been proposed for the task. However, due to the nature of convolution, its receptive field in the time domain is relatively small and the long-range temporal information cannot be captured well. Alternately, due to the sequence processing capability of RNNs, RNNs are naturally appropriate for the task. In [16], LSTM (long short-term memory) is used to process the sequence information. However, for the conventional RNNs, including the simple RNN and LSTM, they usually suffer from the gradient vanishing and exploding problem or gradient decay over layers due to gates with nonsaturated activation functions. Especially for the smartphone-sensors-based activity recognition, a model with long-range processing capability is highly desired.

To address this long-range temporal processing problem, in this paper, we developed a framework of combining short-term spatial/frequency feature extraction and long-term IndRNN recognition model. The contributions of this paper can be summarized as follows.

- A framework of combining short-term spatial and frequency domain feature extraction and long-term independently-recurrent-neural-network (IndRNN)-based recognition is proposed. The long-range temporal processing problem is divided into two problems to take advantage of the periodic characteristics of the sensor data.
- A dense IndRNN model is developed to capture the long-term temporal information. Due to the capability of IndRNN in constructing deep networks and processing long sequences, the dense IndRNN model can effectively process the short-term features to obtain long-term information.

Preprocessing of derotating the sensor data to the world coordinate system and postprocessing of transfer learning to new users in the test set are also used in the proposed method. Experimental results show that the proposed method achieves state-of-the-art performance in the category of single-model-based methods. An earlier version of the proposed method has appeared at a workshop paper for SHL Challenge 2020 [17]. This paper further made a significant improvement by adding a detailed explanation of the proposed method and a thorough analysis of the experiments with ablation study on the models and parameters. Moreover, feature augmentation with temporal changes is further developed, which improves the performance over the earlier one.

The rest of this paper is organized as follows. In Section 2, the related work is described. The proposed method is presented and explained in Section 3. The experimental results and analyses are provided in Section 4. Section 5 concludes the paper.

## 2. Related Work

Vision-based human activity recognition has been widely studied for decades, with many methods proposed in the literature. Environmental sensors such as cameras may become inconvenient in the open or crowded area to gather activity information of each individual. The distance between humans and devices also affects the quality of signals, leading to differences in recognition accuracy. To address these issues, especially to collect the daily activity information based on each individual in all areas, wearable sensors have become an attractive option. Some earlier wearable sensors, requiring markers on people, were intrusive and made people uncomfortable. However, with the quick popularization of the smartphone, smartphone-sensors-based human activity recognition is gaining interest since it does not require further devices other than the smartphone (most people already carry them during the day). Many studies have been conducted for the activity recognition tasks based on smartphone sensors including recognizing indoor activities [8], nursing activities to better care patients [18], and also movements that people perform on their smartphones like typing and scrolling [19]. Different approaches have been proposed for the smartphone-sensors-based activity recognition, including the conventional handcrafted-features-based and the deep-learning-based methods, which will be briefly described in the following subsections.

### 2.1. Conventional Handcrafted-Features-Based Methods

In the conventional handcrafted-features-based methods, spatial/temporal and frequency features are first extracted using techniques including statistical features such as mean, variance, standard deviation, maximum value, minimum value, energy, entropy and Fourier transform spectra. Such features are engineered to capture the information over the sensor data. Alternately, there are also automatic feature extraction methods developed in the literature [20,21]. Automatic feature extraction usually starts with a massive calculation of all potentially useful features from the data, such as statistical features, first-derivative features and fast-Fourier-transform (FFT)-derived features [20]. Algorithms are then designed to perform automatic feature selection, retaining the most useful features for subsequent processing. In [20], a large number of statistical features were first extracted without screening. On this basis, delta series, first-derivative features and FFT-derived features were further extracted and merged with original features together. Feature importance was calculated based on an ERT (Extremely Randomized Trees) classifier and those with low importance were discarded with a diversified forward–backward (DFB) process. Finally, the “best” features were used for classification. Christ et al. [21] constructed a Python package called tsfresh, which first automatically calculates 794 time series features, then automatically filters and extracts them. However, due to the huge amount of computation, it runs slowly. For example, it takes about 30 s to extract the information from a 5 s window. When the dataset is large or the time requirement is strict, it may not be appropriate.

After features are extracted, some conventional machine learning methods such as decision trees [22], *k*-NN (*k*-nearest neighbors) [23], hidden Markov chain [24] and support vector machine (SVM) [25] can be used for the classification of the activity. In [22,23], *k*-NN and decision trees are used as classification models and the abovementioned spatial and frequency domain features are selectively used as input. In [26], a “one-versus-one” SVM is used to perform pairwise combinations selection and a Gaussian kernel is applied to process the features in a high-dimensional space. In [27], random forest is used to predict the activity category of each frame first. Then, activities are smoothed over time with hidden Markov chain considering that the activities in daily life are continuous.

### 2.2. Deep Learning Based Methods

With the increasing applications and success of deep learning in many research areas, deep learning including both CNN and RNN has also been explored to perform the smartphone-sensors-based activity recognition. For the CNN-based methods, Zeng et al. [28] and Zheng et al. [29] used just one convolution layer as a spatial feature extractor to obtain the features at each time step, and then pooling was applied in the time direction to summarize the temporal information. However, with the shallow network and simple temporal processing technique, they could not extract high-level spatial–temporal features and did not achieve high accuracy. Charissa et al. [30] employed a CNN using filters with a large time span to explore the long temporal correlation, and pooling over time was gradually used, alternating with convolutional layers to reduce the loss over time. Zhu et al. [15] proposed use of a 1D DenseNet model in order to take advantage of deeper CNNs. The DenseNet is first applied on each sensor independently and then combined together. All the data in the time domain are sampled and provided as one input to the network to explore the temporal information better. Considering the large volume of the temporal data, this also results in a large number of parameters. Choi et al. proposed an EmbraceNet [14] to fuse multiple CNN models. It also processes each sensor independently and then combines them. In all, the CNN-based methods usually process the temporal sequence with pooling or convolution, which is not effective in the long-range problem.

Since the smartphone-sensors-based human activity recognition is a temporal sequence processing task, RNN can be naturally selected with its temporal processing capability. Francisco et al. proposed a deep framework [16] using convolution and LSTM (long short-term memory) together where the convolution extracts the spatial feature and LSTM helps learn the long-term temporal information. However, the gate mechanism in LSTM makes it difficult to construct deep networks. Some researchers migrate the dense and residual architecture to LSTM to assist in constructing deep networks, but the performance of improvement is not significant [31]. In [32], Rui et al. first used dilated convolutional neural networks to extract local short-term features. Then, a shallow dilated SRU is developed to model the long temporal dependencies. In a word, the conventional RNN models used for classification are usually shallow and cannot effectively construct deep models due to the gradient decay within each layer. On the contrary, the recently proposed IndRNN [33,34] has been shown to be able to better explore the high-level and long-term information, which has also been used in the last two years’ SHL Challenge [35,36] as the base module with only the spatial information or FFT magnitudes using a relatively shallow network. This paper further proposes a framework of combining short-term spatial and frequency features and long-term deep dense IndRNN models for activity recognition.

## 3. Proposed Method

### 3.1. Overall Framework

**Figure 1:**
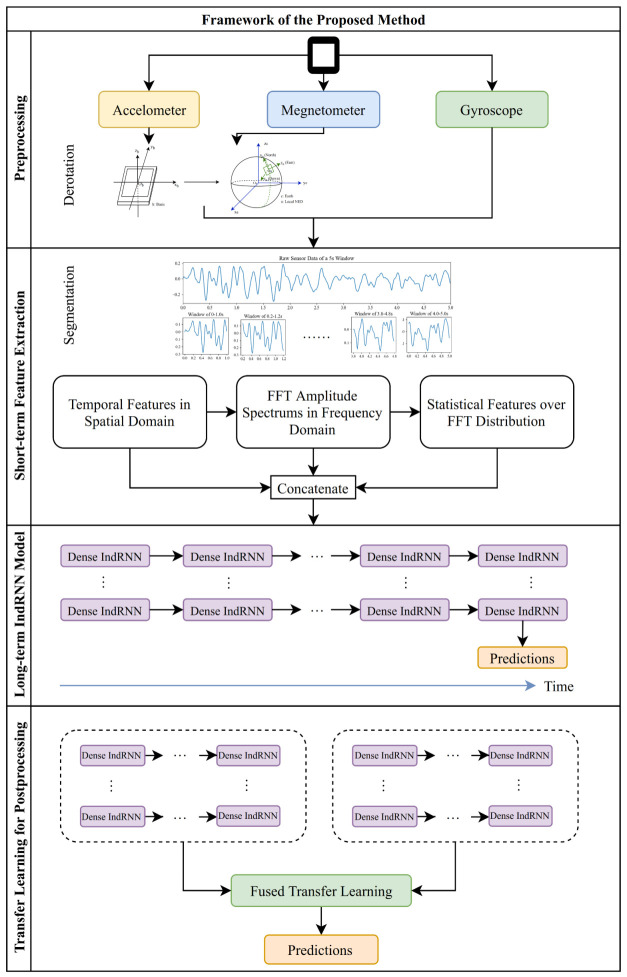
Framework of the proposed method.

This paper proposed an independently-recurrent-neural-network-based long-term activity recognition method based on short-term spatial and frequency domain features. The framework of the proposed method consists of four modules as shown in Figure 1: preprocessing, short-term spatial and frequency feature extraction, long-term IndRNN model and transfer learning for postprocessing. The preprocessing and short-term feature extraction modules process the input data to short-range spatial features and frequency domain features to accommodate the periodic nature of the smartphone sensors data. Then, the IndRNN model, taking advantage of its ability to process long sequences and construct deep models, is applied as the main recognition model to solve the long-range classification problem. Finally, transfer learning is adopted as postprocessing to fine-tune the model in order to realize user independence. Details on each module are presented in the following.

### 3.2. Preprocessing

For the current smartphones such as HUAWEI Mate 9 used to collect data in the SHL dataset [13,37], the sensor data are measured in a coordinate according to the smartphone position. The basis of triaxial sensors is $\left(x_{b},y_{b},z_{b}\right)$ where, for most phones, $x_{b}$ is along the shorter side and pointing right, $y_{b}$ is along the longer side and pointing up and $z_{b}$ is perpendicular to the screen and pointing out. The accelerometer and magnetometer sensors, two of the smartphone sensors, measure the device’s acceleration and the magnetic field of the Earth at the device location, respectively. They are represented by two three-dimensional vectors, representing the acceleration of the phone and the magnetic field of where the phone is, respectively. Since the data are measured in the coordinate according to the smartphone position, the sensor data can be inconsistent in the world coordinate when only the phone is rotating without the user’s body movement. In turn, it will affect the classification accuracy of the user’s activity without preprocessing. Therefore, to reflect the user’s real movement in the world coordinate, the sensor data need to be derotated to the consistent world coordinate system.

**Figure 2:**
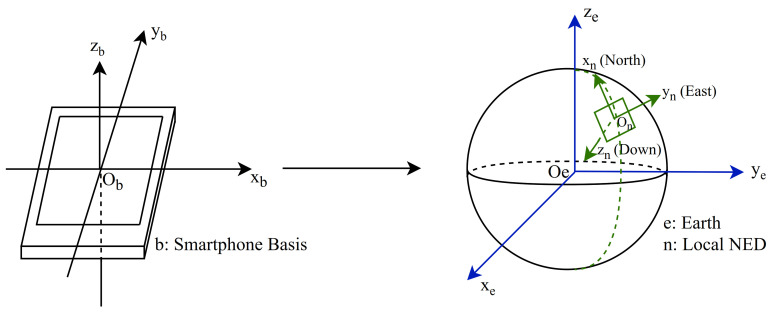
Derotation of coordinates from the smartphone coordinate system to the NED (north-east-down) coordinate system.

In this paper, the NED (north-east-down) coordinate system is used to transform the sensor data as shown in Figure 2, where $x_{n}$ points toward east, $y_{n}$ points toward magnetic north and $z_{n}$ points up toward the sky. The transform can be performed by multiplying the raw sensor data with the rotation matrix *R* derived from the orientation sensor of the device in quaternions $\left[q_{w},q_{x},q_{y},q_{z}\right]$ as shown in Equations (1) and (2).

(1) $$R=\left[\begin{array}{ccc} 1-2\left(q_{y}^{2}+q_{z}^{2}\right) & 2\left(q_{x}q_{y}-q_{w}q_{z}\right) & 2\left(q_{x}q_{z}+q_{w}q_{y}\right)\\ 2\left(q_{x}q_{y}+q_{w}q_{z}\right) & 1-2\left(q_{x}^{2}+q_{z}^{2}\right) & 2\left(q_{y}q_{z}-q_{w}q_{x}\right)\\ 2\left(q_{x}q_{z}-q_{w}q_{v}\right) & 2\left(q_{v}q_{z}+q_{w}q_{x}\right) & 1-2\left(q_{x}^{2}+q_{v}^{2}\right) \end{array}\right]$$

(2) $$\left[\begin{array}{c} x_{n}\\ y_{n}\\ z_{n} \end{array}\right]=R\left[\begin{array}{c} x_{b}\\ y_{b}\\ z_{b} \end{array}\right]$$

where $\left(x_{n},y_{n},z_{n}\right)$ represents the transformed data in the NED coordinate system, which is consistent with the user’s movement. The transformed data can then be used for the following feature extraction.

### 3.3. Short-Term Spatial and Frequency Domain Feature Extraction

For the sensors used in the HUAWEI Mate 9, the sampling rate is 100 Hz. Data from a window of 5 s are used for each classification, resulting in 500 frames of data. Note that the proposed method can work with samples of any temporal window, but data of the 5 s window are provided in the SHL dataset. Generally, processing long-range data such as 500 steps is difficult due to the complex temporal pattern. The data from the smartphone sensors have also been shown to be periodic [11]. Therefore, some short-term spatial and frequency domain features are extracted first as explained following.

**Figure 3:**
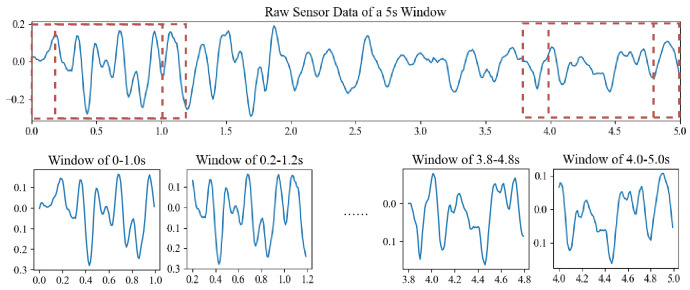
Illustration of the short-term data segmentation.

First, the data of each 500-frame (5 s) sample were segmented into 21 windows of 100-frame (1 s) overlapping sliding windows as shown in Figure 3. Each segmented window contains short-term signals and long-time signals can be obtained by combining them over time. The data from seven sensors—accelerometer, gyroscope, magnetometer, linear acceleration, gravity sensor, orientation sensor and ambient pressure sensor—are provided for classification, resulting in a total of 20 channels of data. Since the accelerometer is a superposition of the linear acceleration and gravity, the linear acceleration and gravity data are not used to reduce the size of the data input. Since orientation is used to derotate the other sensors’ data, it is no longer used after the preprocessing. In all, the data from the gyroscope, derotated data from the accelerometer and magnetometer and pressure are used in our method, which contains 10 channels.

**Figure 4:**
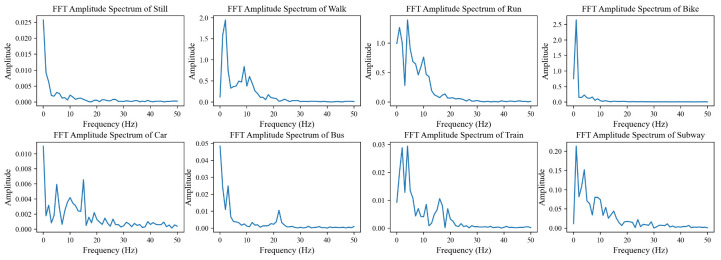
Example fast Fourier transform (FFT) amplitude spectra from one segmented window of different classes.

For each segmented window, some spatial features over time are first extracted, including mean, numbers above mean, numbers below mean, standard deviation, minimum value and maximum value, similarly as in [19]. Moreover, for pressure, the data are normalized per sample and used as input to show the change within each sample. The pressure data did not perform well in activity recognition but did perform well in the location recognition model introduced later. The description of the features is shown in Table 1. Alternately, due to the strong periodicity of the smartphone sensor data, fast Fourier transform (FFT) is used to transform the data into the frequency domain. The FFT amplitude spectra are then extracted as features where only the magnitudes of the coefficients are used (half of the total data). Some examples of the FFT amplitude spectra from all the classes are shown in Figure 4. It can be seen that the distribution of FFT amplitude spectra can be quite different among different classes. Therefore, in addition to the amplitude spectrum, some statistical features on top of the frequency features including mean and standard variation are also extracted and combined with previous features.

### 3.4. Long-Term IndRNN (Independently Recurrent Neural Network) Model

With the short-term spatial/temporal and frequency domain features extracted, a long-term recognition model is further proposed for the final recognition. In this paper, our previously proposed independently recurrent neural network (IndRNN) [33,34] is adopted as the basic model. The structure of the IndRNN [33,34] is as follows:

(3) $$h_{t}=\sigma \left(Wx_{t}+u⊙h_{t-1}+b\right)$$

where $x_{t}\in R^{M}$ and $h_{t}\in R^{N}$ are the input and hidden state at time step t, respectively. $W\;\in \;R^{M\times N},u\;\in \;R^{N}$ and $b\in R^{N}$ are the weights for the current input and the recurrent input and the bias of neurons. ⊙ represents the Hadamard product and σ is the nonlinear activation function of neurons. N is the number of neurons of this IndRNN layer. With this form, neurons in IndRNN are independent from one another and the gradient backpropagation can be calculated for each of them. Accordingly, by regulating the recurrent weights, it sufficiently addresses the gradient vanishing and exploding problems. Therefore, it can process long sequences. It can also work robustly with nonsaturated functions such as ReLU; thus, it is able to construct deep networks.

**Figure 5:**
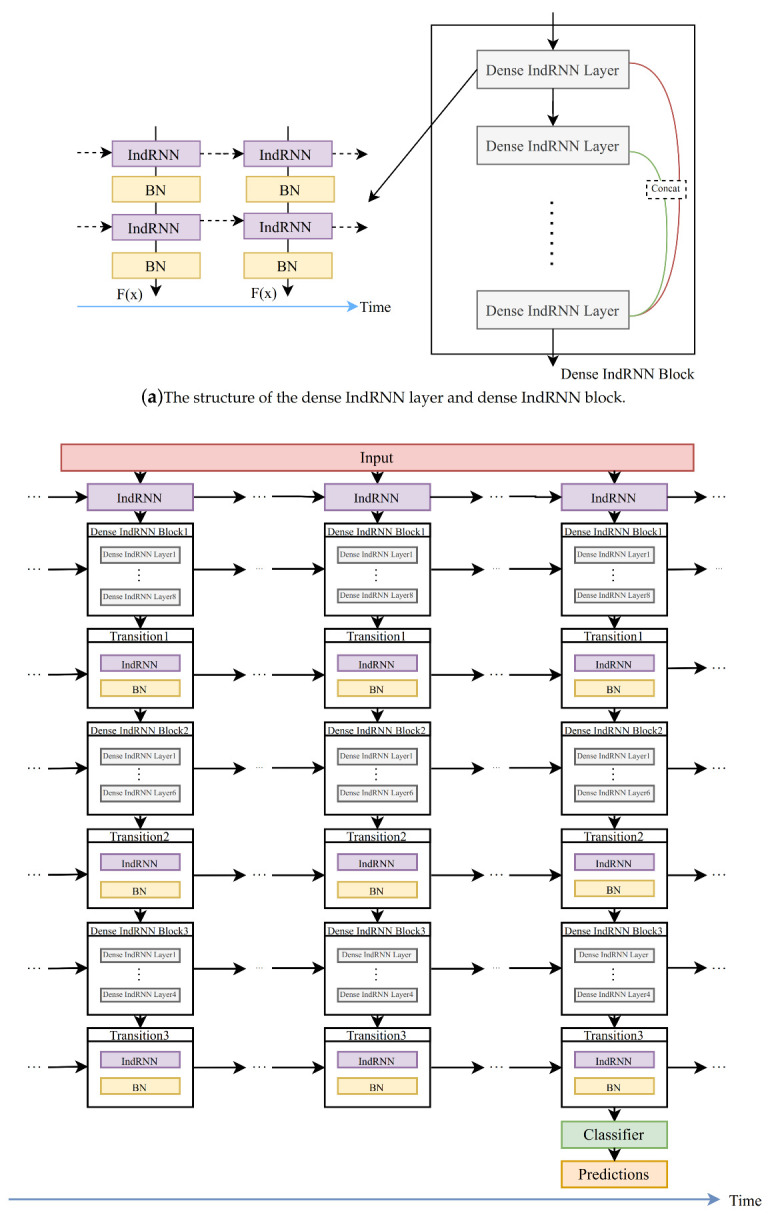
Illustration of the proposed dense IndRNN structure.

In this paper, we propose the use of a deep dense IndRNN as the main classification model. The diagram of the proposed dense IndRNN model is shown in Figure 5b and the detailed illustration of each dense layer and dense block is shown in Figure 5a. The overall architecture follows [34]. It consists of three dense blocks with 8, 6 and 4 dense layers, and each dense layer contains two IndRNNs as shown in Figure 5b. Batch normalization is used after each IndRNN layer to accelerate training. Dense architecture concatenates feature output from all the previous dense layers in a dense block as the input for the next dense layer. It facilitates the feature reuse of the relatively shallow layers. After each dense block, a transition block with one IndRNN layer is followed to compress the features as a bottleneck, where the outputs are usually reduced to half of the input features. Finally, a classifier with one linear function and softmax activation is used at the last time step for the final classification.

The cross-entropy loss is used as the objective function for training, which is

(4) $$L=\sum_{i=1}^{8}t_{i}\log\left(p_{i}\right)$$

where $t_{i}$ is an indicator variable, which is equal to 1 when the prediction is right and is equal to 0 when the prediction is wrong. $p_{i}$ is the predicted probability of this sample. The categorical cross-entropy has been widely used for classification.

### 3.5. Transfer Learning for Postprocessing

Different activities can be classified with the above preprocessing, short-term feature extraction and long-term IndRNN-based recognition. However, considering that the smartphone can be placed at any location by the user such as holding in the hand, bag or in the lap pocket, large differences in the sensor data can exist. Directly classifying different sensor data captured from different locations can be difficult, and the most appropriate features used for classification under different locations may also be different. Therefore, considering the differences among different sensors, the location of the sensor data is first recognized. Then, in the test, we can pinpoint the location of the data and use an appropriate model for classification. In this process, the labels of the sensor data are changed to the locations of the sensors. A simple plain IndRNN model of stacking six-layer IndRNNs is used for the classification.

**Figure 6:**
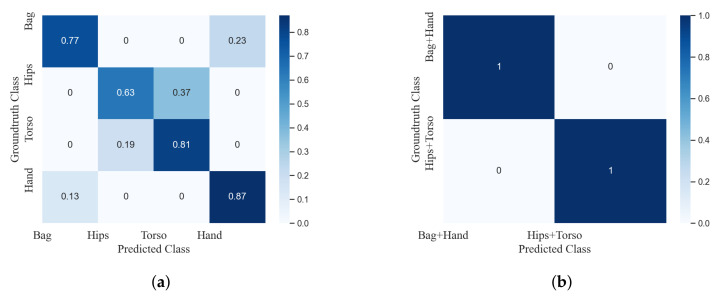
Confusion matrices of the location recognition on the validation set. (**a**) Confusion matrix of the location recognition on the validation set: on four locations. (**b**) Confusion matrix of the location recognition on the validation set: two groups—Bag and Hand, Hips and Torso.

The location recognition result in terms of the confusion matrix is shown in Figure 6a, where four locations are used, including bag, hips, torso and hand. It can be observed that while different locations can be recognized with relatively good accuracy, there is still some confusion among different classes, especially between bag and hand and between hips and torso. If locations are recognized into two groups, bag and hand as one group and hips and torso as the other group, the classification of two groups can be accurate, as shown in Figure 6b. It indicates that the features of the data from each group can be similar while the features from different groups can be distinguished. Therefore, in the proposed scheme, group-based location recognition is used; the data are first classified into two groups and then further recognized as different activities. Note that in the SHL dataset used in the experiment, all of the data from the test set come from one unknown location; thus, they are classified first to one location group and only one model is constructed for this recognized location group.

**Figure 7:**
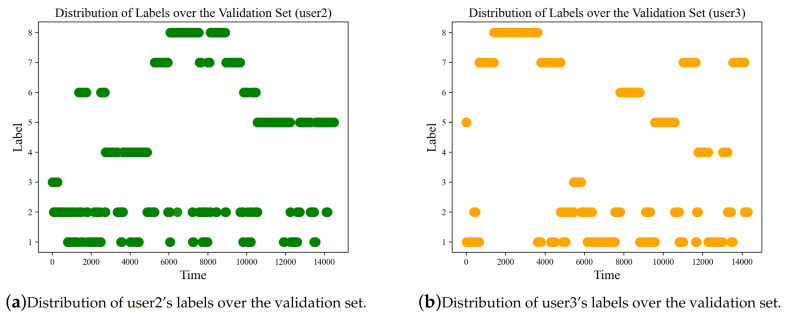
Distribution of labels over the validation set.

Alternately, due to the limitation of the dataset, which only contains data from three users (although with a large amount of data—196,072 frames), transfer learning is used to generalize the model to different users quickly. In the SHL dataset, only user1 is used as training data, a small amount of data from the other two users are used as validation data and the remaining data from user2 and user3 are kept for testing. To fully take advantage of the validation data (which is allowed in the challenge), the validation data are first split and part of it is used to transfer the model learned on the training data of user1 to the test data of user2 and user3. For simplicity, the learned model is directly fine-tuned on the transfer data. The most common way of transfer learning is to use a half of the validation set as transfer training set and another half acts as transfer validation set. However, in this challenge, splitting the validation set directly into parts may lead to overfitting because labels of the validation set distribute unevenly as shown in Figure 7. Therefore, the data with the same labels are first stacked together, then divided with a similar proportion of data from all the classes to construct the transfer training set and the transfer validation set for the transfer learning process.

**Figure 8:**
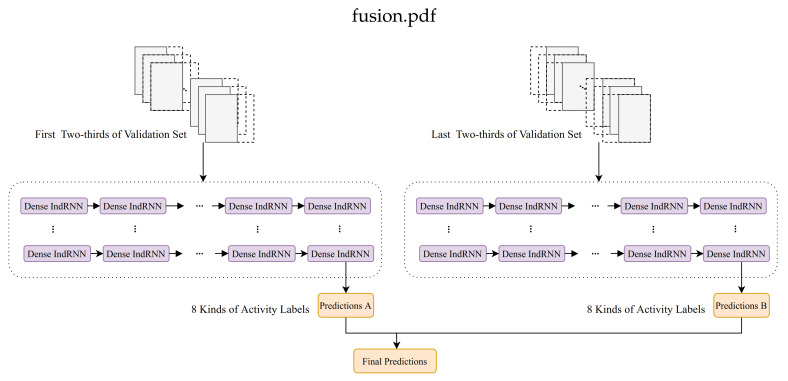
Diagram of the fused transfer learning.

When conducting the transfer learning process, it leads to different accuracies using the first half and the second half of the original validation set for training because of the limited size of the validation set. Accordingly, we further swap the transfer training and transfer validation set to learn two models, noted as TransferA and TransferB, and then fuse them to take advantage of all of the data. The diagram of the transfer learning is shown in Figure 8.

## 4. Experimental Results

### 4.1. Dataset and Setup

#### 4.1.1. SHL Dataset

The SHL dataset [13,37] is used for evaluation in this paper, which is also the dataset used in the SHL Challenge 2020. It was recorded over seven months in 2017 from three users (user1, user2 and user3). The goal of this dataset is to use machine learning methods and heuristics to realize the recognition of users’ eight locomotion modes and transportation (Still, Walk, Run, Bike, Bus, Car, Train and Subway). The smartphone used to collect data is put on four locations on the body (Bag, Hips, Torso and Hand). The dataset is used with the aim to realize user independence and location independence. To be specific, the training set contains 272×4 h from four locations of user1. The validation set consists of 40×4 h of data from four locations of the combination of user2 and user3. The test set contains 160 h of data of user2 and user3 from an unknown location (Hips after the Challenge result is published).

The data are collected from seven raw sensors—accelerometer, magnetometer, gyroscope, magnetometer, linear acceleration, gravity sensor, orientation sensor and ambient pressure sensor—with a combined total of 20 channels. The sampling rate is 100 Hz; all of the data are segmented into 5 s windows and all of the 5 s windows are shuffled in time. The data sizes of the training set, validation set and test set are 196,072×500, 28,789×500 and 57,573×500, respectively.

#### 4.1.2. Training Setup

For training, Adam [38] is used for optimization. The learning rate of our model is set to $2\times 10^{-4}$ at first. To restrain the slightly larger fluctuation at the beginning of the training process, it is set to $2\times 10^{-5}$ at the first 10 epochs as a learning rate warmup strategy. The learning rate drops 10 times once the validation accuracy does not increase (over a large patience of 100). A minibatch with batch size of 128 is used to train our model. The dense block configuration is set to (8, 6, 4), where in the first, second and third dense block, 8, 6 and 4 dense layers are used, respectively. This keeps a relatively similar number of neurons in each dense block. The growth rate is set to 48.

In our model, ReLU is applied as an activation function. Compared to the tanh and sigmoid function, it not only reduces the amount of computation but also helps to alleviate the problem of gradient vanishing. In order to reduce overfitting, dropout is applied after the input (0.5), each dense layer (0.5), each bottleneck layer (0.1) and each transition layer (0.3).

#### 4.1.3. Evaluation

The final performance is evaluated using the F1 score. Traditionally, the F1 score is used in evaluating binary classifications and can be defined with precision and recall as follows:

(5) $$\begin{array}{c} \mathrm{Precision}=\frac{TP}{TP+FP}\\ \mathrm{Recall}=\frac{TP}{TP+FN} \end{array}$$

where TP represents true positive (the number of items correctly labeled as belonging to the positive class), FP is false positive (the number of items incorrectly labeled as belonging to the class) and FN is false negative (items which are not labeled as belonging to the positive class but should have been). Among them, precision focuses on assessing how much of all the data that are predicted to be positive are true positive. Recall focuses on how many samples are successfully predicted to be positive among those that are real positive. In multicategory classification, the precision and recall are calculated for each class separately, and the overall precision, recall and F1 score can be obtained as follows:

(6) $$\begin{array}{c} \mathrm{Precision}=\frac{P_{still}+\ldots +P_{subway}}{8}\\ \mathrm{Recall}=\frac{R_{still}+\ldots +R_{subway}}{8}\\ \;F1\;\mathrm{score}=\frac{2\times \;\mathrm{Precision}\;\times \;\mathrm{Recall}\;}{\;\mathrm{Precision}\;+\;\mathrm{Recall}\;} \end{array}$$

F1 score is a measurement that combines precision and recall by calculating the harmonic mean of them. When they are close, F1 score is approximately the average of the two. For the case of two numbers, it coincides with the square of the geometric mean divided by the arithmetic mean. It can better present the results, especially in the case of imbalanced data distribution among different categories.

In the SHL Challenge, since the location is unknown, location recognition is first performed to recognize the location of the test set. In this paper, since the location is already reported, the validation data from the known location (Hips) are used for validation. It is observed that there is no large difference using a group-based location or a specific location. In the practical applications, we argue that the locations are always unknown and the group-based location may better describe the data as shown in Figure 7.

### 4.2. Ablation Studies on Models, Augmentation and Learning Rates

**Figure 9:**
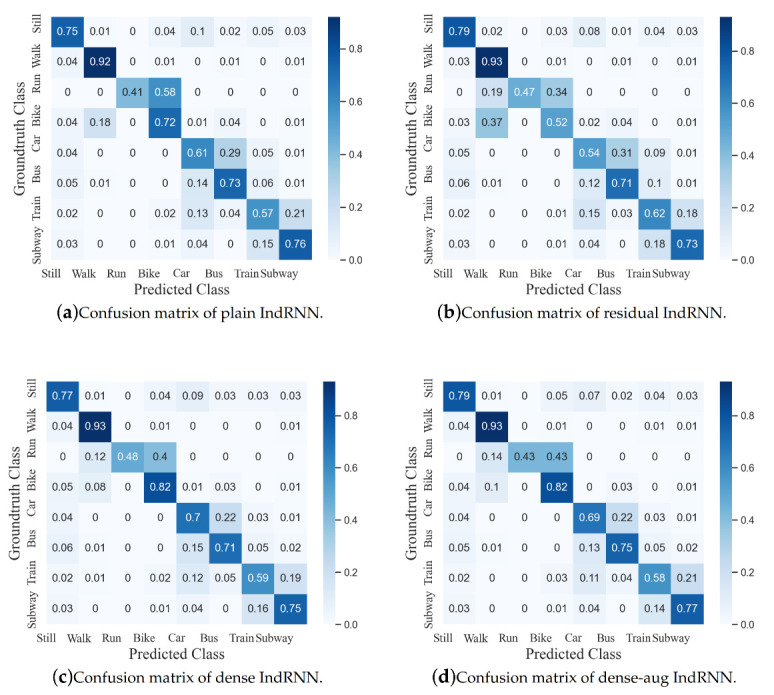
Confusion matrices of different IndRNN architectures.

First, three different model architectures are evaluated including the plain IndRNN, residual IndRNN and dense IndRNN. The results from the test set are shown in Table 2 and the confusion matrices are shown in Figure 9. It can be seen that the dense IndRNN performs the best. Therefore, in the following experiments, dense IndRNN is used as the baseline of the model.

### 4.3. Transfer Learning

Alternately, feature augmentation is also explored in the proposed method. In addition to the input data and features at each time step for input of the network and deeper layers of the network, this paper also augments the input data and features with the temporal difference. The augmentation can be viewed as a form of optical flow in the video-based classification tasks. It provides the first-order change information for better processing. This presents the network with a direct variation over time to better capture the temporal patterns. The result is also shown in Table 2, and it can be seen that the feature augmentation improves performance.

**Figure 10:**
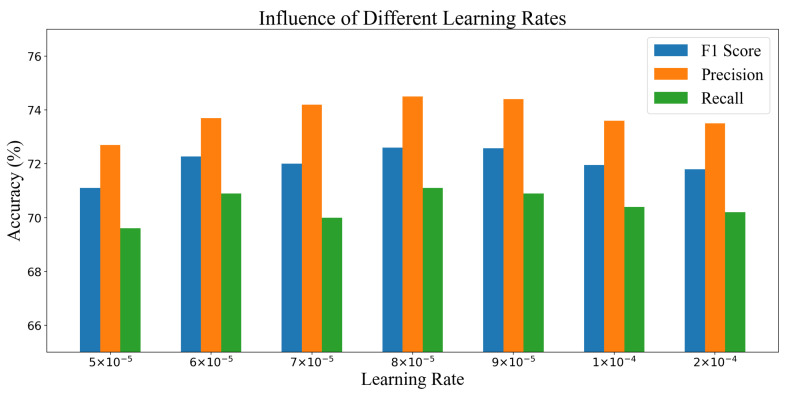
Illustration of using different learning rates.

Considering the large differences between the training data and validation/test data (from different users), the learned model tends to become overfitting when the learning rate is too small. Therefore, the effects of different learning rates are further studied on the final performance. The results are shown in Figure 10. It can be seen that the network performs similarly in a wide range of learning rates. The learning rate is set to $8\times 10^{-5}$ in the experiments.

The dense IndRNN model trained above with the feature augmentation and the learning rate is used for the transfer learning [39] to further improve the performance on the final test dataset as described in Section 3.5. The learning rate in the transfer learning is set to $2\times 10^{-5}$ in training empirically. In this paper, the simple fine-tuning of the model on the transfer learning sets is used. The result is shown in Table 3. It can be seen that after transfer learning, the accuracy of validation set increases to 80.72%, which means that cross-user transfer learning is useful for testing on the data from different users. It is noticed that the performance of the TransferB model is better than that of the TransferA model, which is due to the uneven distribution of the two transfer learning datasets.

**Figure 11:**
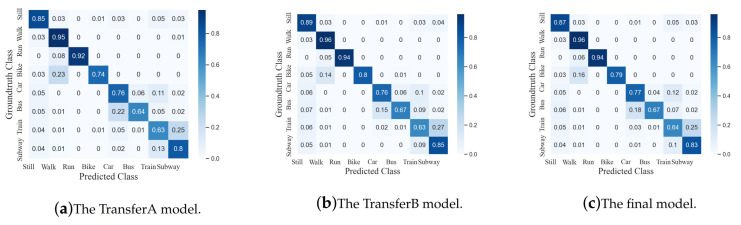
Confusion matrices of the different transfer models.

By comparing the confusion matrices before and after transfer learning shown in Figure 9 and Figure 11, it can be further observed that the recognition accuracy increases greatly for most classes (except Bike and Bus). For Still, transfer learning further brings an accuracy improvement around 6%, which eliminates the mistakes of being predicted as Bike, Car or Bus. For Walk, the accuracy increases around 3%, mainly reducing the confusion with Train or Subway. Moreover, the accuracy improvement for Run is significant, from 43% to 94%. Before transfer learning, over 40% of Run samples were predicted as Bike, while after that, it largely improved. It indicates that the activity Run is of strong user dependence. The recognition accuracies of three motor-powered activities, including Car, Train and Subway, also improved while Bus slightly decreased and was misclassified as Car. While the proposed method achieves a relatively high performance on the other locomotions, the accuracies of the four motor-powered activities are still relatively low due to their strong similarities. Methods on distinguishing the small differences among them are highly desired, which will be investigated in the future.

### 4.4. Comparison with State-of-the-Art Classification Methods

The proposed method is further compared with the existing methods [40,41,42,43,44,45,46,47,48,49,50,51]. The results are shown in Table 4, including comparisons with the existing machine learning and deep learning methods. It can be seen that the proposed IndRNN long-term temporal recognition greatly improves the performance over other single-model based machine learning and deep learning methods. However, it is slightly worse than the model fusion method of DenseNetX + GRU [40] (the first place of the SHL Challenge 2020), which fuses the CNN and RNN models together and also fuses the features of each sensor processed individually. It indicates that the spatial processing and effective combination of all the sensors may be important for the recognition. On the other hand, the proposed IndRNN model can also be equipped with enhanced spatial processing and combination of sensors to further improve the performance, which will be studied in the future.

## 5. Conclusions

In this paper, we presented a framework of combining short-term spatial/frequency feature extraction and long-term IndRNN model for smartphone-sensors-based activity recognition. The short-term spatial and frequency domain features are first extracted with the Fourier transform to deal with the periodic nature of the sensor data. With the conventional statistical features, the FFT amplitude spectra and the statistical features of the FFT spectra are extracted to characterize the data of a short-term window. Then, a dense IndRNN model is further developed to learn the long-term temporal features on top of the short-term spatial and frequency domain features. Finally, transfer learning is adopted in the experiments to realize the user independence, which further improves the performance on the test set. Experiments show that our model achieved an accuracy of 80.72% on the SHL dataset, which is better than the existing single-model-based methods. 

## Figures and Tables

**Table 1:** Extracted short-term features in the spatial–temporal domain and their definitions.

Time Domain Features	Description
Mean	The average value of the data for each axis in the window
Numbers above Mean	The numbers of values above the mean of the window
Numbers below Mean	The numbers of values below the mean of the window
Standard Deviation	Standard deviation of each axis in the window
Minimum Value	The minimum value of the data for each axis in the window
Maximum Value	The maximum value of the data for each axis in the window
Per Sample Normalized Pressure	The normalized pressure of each sample

**Table 2:** Results on using different model architectures and augmentation.

Model	Presicion	Recall	F1 Score
Plain IndRNN	71.70%	67.40%	69.48%
Residual IndRNN	70.17%	65.93%	67.98%
Dense IndRNN	74.37%	69.41%	71.80%
Dense-IndRNN-aug	76.25%	72.00%	74.06%

**Table 3:** Results of the different transfer learning models.

Method	Performance	Final Performance
TransferA	78.11%	80.72%
TransferB	80.97%	

**Table 4:** Results of the proposed method in comparison with the existing methods.

Method	Performance
XGBoost [48]	55.0%
Nearest neighbor smoothing [46]	61.2%
Random forest (without location estimation) [45]	62.6%
Random forest (with location estimation) [44]	69.1%
XGBoost (semisupervised) [41]	77.9%
GAN [51]	34.4%
Multiview CNN [50]	37.3%
Logistic regression [47]	55.7%
InceptionTime [43]	69.4%
Three-layer CNN [42]	76.4%
CNN + LSTM [49]	52.8%
DenseNetX + GRU (Model Fusion based) [40]	88.5%
Dense-IndRNN-aug	80.7%
